# Neonatal Mortality of Planned Home Birth in the United States in Relation to Professional Certification of Birth Attendants

**DOI:** 10.1371/journal.pone.0155721

**Published:** 2016-05-17

**Authors:** Amos Grünebaum, Laurence B. McCullough, Birgit Arabin, Robert L. Brent, Malcolm I. Levene, Frank A. Chervenak

**Affiliations:** 1 Department of Obstetrics and Gynecology, Weill Medical College of Cornell University, New York, New York, United States of America; 2 Center for Medical Ethics and Health Policy, Baylor College of Medicine, Houston, Texas, United States of America; 3 Center for Mother and Child, Philipps University, Marburg, Germany and Clara Angela Foundation, Berlin, Germany; 4 Thomas Jefferson University, Alfred I. DuPont Hospital for Children, Wilmington, Delaware, United States of America; 5 Division of Pediatrics & Child Health, University of Leeds, Leeds, United Kingdom; University of Washington, UNITED STATES

## Abstract

**Introduction:**

Over the last decade, planned home births in the United States (US) have increased, and have been associated with increased neonatal mortality and other morbidities. In a previous study we reported that neonatal mortality is increased in planned home births but we did not perform an analysis for the presence of professional certification status.

**Purpose:**

The objective of this study therefore was to undertake an analysis to determine whether the professional certification status of midwives or the home birth setting are more closely associated with the increased neonatal mortality of planned midwife-attended home births in the United States.

**Materials and Methods:**

This study is a secondary analysis of our prior study. The 2006–2009 period linked birth/infant deaths data set was analyzed to examine total neonatal deaths (deaths less than 28 days of life) in term singleton births (37+ weeks and newborn weight ≥ 2,500 grams) without documented congenital malformations by certification status of the midwife: certified nurse midwives (CNM), nurse midwives certified by the American Midwifery Certification Board, and “other” or uncertified midwives who are not certified by the American Midwifery Certification Board.

**Results:**

Neonatal mortality rates in hospital births attended by certified midwives were significantly lower (3.2/10,000, RR 0.33 95% CI 0.21–0.53) than home births attended by certified midwives (NNM: 10.0/10,000; RR 1) and uncertified midwives (13.7/10,000; RR 1.41 [95% CI, 0.83–2.38]). The difference in neonatal mortality between certified and uncertified midwives at home births did not reach statistical levels (10.0/10,000 births versus 13.7/10,000 births p = 0.2).

**Conclusions:**

This study confirms that when compared to midwife-attended hospital births, neonatal mortality rates at home births are significantly increased. While NNM was increased in planned homebirths attended by uncertified midwives when compared to certified midwives, this difference was not statistically significant. Neonatal mortality rates at home births were not significantly different in relationship to professional certification status of the birth attendant, whether the delivery was by a certified or an uncertified birth attendant.

## Background and Objectives

Over the last decade, planned home births in the United States (US) have increased,[1] and have been associated with increased neonatal mortality and other morbidities.[2–8] The majority of planned home births in the US are attended by midwives which on United States birth certificate data are identified by professional certification status as either certified nurse midwives (CNM), nurse midwives certified by the American Midwifery Certification Board, and “other” or uncertified midwives who are not certified by the American Midwifery Certification Board. These uncertified midwives include “certified professional midwives” (CPM), direct entry midwives, and lay midwives (Table 1). The American College of Obstetrics and Gynecology and the American Academy of Pediatrics list certification by the American Midwifery Certification Board (AMCB) as a requirement for midwives attending home births. [9,10]. Nonetheless, about 2/3 of all planned home births are attended by uncertified birth attendants.[11]

**Table 1:** Differences between American Midwifery Certification Board (AMCB) Certified and Uncertified Midwives.

Title and required Academic Degree	AMCB Certification	Description
**Certified Nurse-Midwife (CNM):** Graduate Degree	AMCB Certified; Licensed in all 50 states plus the District of Columbia and US territories	An individual trained and licensed in both nursing and midwifery. Nurse-midwives possess at least a bachelor’s degree from an accredited institution of higher education and are certified by the American College of Nurse Midwives.
**Certified Midwife (CM):** Graduate Degree	AMCB Certified; Licensed in New Jersey, New York, and Rhode Island. Authorized by permit to practice in Delaware	An individual trained and certified in midwifery. Certified midwives possess at least a bachelor’s degree from an accredited institution of higher education and are certified by the American College of Nurse Midwives.
**Certified Professional Midwife (CPM):** High School Diploma or Equivalent	AMCB Uncertified; Regulated in 28 states (variously by licensure, certification, registration, voluntary licensure, or permit)	An individual trained in midwifery who meets practice standards of the North American Registry of Midwives. Two primary pathways: Portofolio evaluation process, no degree or diploma required. OR Accredited formal education pathway, high school diploma required
**Direct-Entry Midwife (DEM):** No degree required	AMCB Uncertified	An independent practitioner educated in midwifery through a variety of sources that can include: self-study, apprenticeship, a midwifery school, a college or university program
**Lay Midwife:** No degree required	AMCB Uncertified	An individual who is not certified or licensed as a midwife but has been trained informally through self-study or apprenticeship.

Modified from http://www.amcbmidwife.org/amcb-certification/why-amcb-certification-

http://mana.org/about-midwives/what-is-a-midwife

Last accessed: 5/5/2016

In a previous study we reported that neonatal mortality is increased in planned home births but we did not perform an analysis for the presence of professional certification status. After publication of our study on neonatal mortality in relationship to birth setting, [7] we were asked to provide a separate analysis of outcomes for the disparate midwife groups attending home births to adjust for variations in requirements for certification of midwives attending home births. [12]

The objective of this study therefore was to undertake an analysis to determine whether the professional certification status of midwives or the home birth setting are more closely associated with the increased neonatal mortality of planned midwife-attended home births in the United States.

## Materials and Methods

This study is a secondary analysis of our prior study.[7] The 2006–2009 period linked birth/infant deaths data set was analyzed to examine total neonatal deaths (deaths less than 28 days of life) in term singleton births (37+ weeks and newborn weight ≥ 2,500 grams) without documented congenital malformations by certification status of the midwife: certified nurse midwives (CNM), nurse midwives certified by the American Midwifery Certification Board, and “other” or uncertified midwives who are not certified by the American Midwifery Certification Board. Table 1 describes the differences between AMCB certified nurse midwives and AMCB noncertified “other” midwives.

We excluded infants if they met any of the following criteria: birth attendant type was not recorded; birth place was anywhere else but the hospital or home, or not recorded; gestational age was <37 weeks or not recorded; birth weight was <2500g or not recorded; multiple gestations; any congenital anomaly, Down syndrome, or other chromosomal disorder was confirmed or pending; and a resident of a foreign country.

The 1989 revision of the U.S. Standard Certificate of Live Birth provides additional detail for out-of-hospital births and makes it possible to distinguish among out-of-hospital births at home, in a birthing center, or other specified location.[13]

In contrast to the birth certificate files, which provide information on delivery, the CDC linked birth/infant death data (for live births and infant deaths) allows analysis of neonatal mortality. This data set (linked file) is generally the preferred source for infant and neonatal mortality in the United States (US).[13]

It contains detailed information for the approximately 4 million births in the United States each year, including birth setting, birth attendant, and neonatal mortality. Period linked files use all births in a year as the denominator and all deaths in a year as the numerator, regardless of when the birth occurred (e.g. if the birth was in late 2008 then neonatal death could have been 2008 or 2009 but counted in the 2008 numerator only if the death occurred in 2008).

Certified home birth midwives (RR = 1) served as reference. Data were extracted using SAS 9.3 (Cary, NC) and compiled in Excel. Relative risks (RR) and 95% confidence intervals (95% CI) were computed in SAS 9.3.

Because non-identifiable data from a publicly available data set were used, our study was not considered human subjects research and did not require review by the Institutional Review Board of Weill Medical College of Cornell University.

## Results

Table 2 shows the neonatal mortality (0–27 days) by birth setting, midwife certification status, and by parity and postdates. There were 1,158,548 deliveries between 2006 and 2009 that met study criteria, 1,096,555 in the hospital and 61,993 at home. Uncertified midwives performed 70.3% of all home births (43,604/61,993), as compared to 29.7% (18,389/61,993) which were performed by certified nurse midwives.

**Table 2:** Term neonatal mortality (0–27 days) per 10,000 births by birth setting, midwife certification status, and parity and postdates.

Neonatal Mortality	Per 10,000 (n/total)	RR (95% CI)	P value
**TOTAL**			
Hospital certified nurse midwife (CNM)	3.2 (356/1,096,555)	0.33 (0.21–0.53)	<0.0001
Home certified nurse midwife (CNM)	10.0 (18/18,389)	1	
Home uncertified midwife	13.7 (60/43,604)	1.41 (0.83–2.38)	NS
**Neonatal mortality (para = 0)**			
Hospital certified nurse midwife (CNM)	3.3 (141/432,018)	0.19	<0.0001
Home certified nurse midwife (CNM)	17.6 (7/4,044)	1	
Home uncertified midwife	23.7 (23/9,840)	1.35 (0.58–3.15)	NS
**Neonatal mortality (para>0**)			
Hospital certified nurse midwife (CNM)	3.2 (213/658,272)	0.45	<0.02
Home certified nurse midwife (CNM)	7.3 (10/13,980]	1	
Home uncertified midwife	10.6 (35/33,187)	1.47 (0.73–2.98)	NS
**Neonatal mortality (<41 wks)**			
Hospital certified nurse midwife (CNM)	3.4 (295/873,226)	0.35 (0.20–0.61)	0.0002
Home certified nurse midwife (CNM)	9.8 (13/13,500)	1	
Home uncertified midwife	10.4 (32/30,921)	1.08 (0.56–2.05)	NS
**Neonatal mortality (≥41 wks)**			
Hospital certified nurse midwife (CNM)	2.7 (61/223,329)	0.26 (0.11–0.67)	<0.005
Home certified nurse midwife (CNM)	10.3 (5/4,889)	1	
Home uncertified midwife	21.6 (27/12,683)	2.08 (0.80–5.40)	NS

CNM, certified nurse midwife; MW, midwife, AMCB, American midwife certification board; CI confidence interval; NS, not significant; RR = relative risk.

Neonatal mortality rates in hospital births attended by certified midwives were significantly lower (3.2/10,000, RR 0.33 95% CI 0.21–0.53) than home births attended by certified midwives (NNM: 10.0/10,000; RR 1) and uncertified midwives (13.7/10,000; RR 1.41 [95% CI, 0.83–2.38]). The difference in neonatal mortality between certified and uncertified midwives at home births did not reach statistical levels (10.0/10,000 births versus 13.7/10,000 births p = 0.2).

Neonatal mortality rates in hospital births attended by certified midwives were significantly lower for nulliparous and multiparous women as well as in pregnancies <41 weeks and ≥41 weeks of gestation when compared to neonatal mortality rates at home births attended by uncertified and certified midwives (Nulliparous women: hospital midwives: neonatal mortality 3.3/10,000 versus 17.6/10,000 by certified and 23.7/10,000 for uncertified midwives; p<0.001. Multiparous women: hospital midwives’ neonatal mortality 3.2/10,000 versus 10.6/10.000 for uncertified and 7.3/10,000 for certified midwives; p<0.02.; <41 weeks gestation: hospital certified midwives’ neonatal mortality 3.4/10,000 versus 10.4/10,000 in uncertified midwives and 9.8/10,000 in certified midwives; p = 0.0002; ≥41 weeks gestation: hospital certified midwives’ neonatal mortality 2.7/10,000 versus 21.6/10,000 for uncertified and 10.3/10,000 for certified midwives; p<0.001).

Neonatal mortality rates at home births both for certified and uncertified midwives for nulliparous women were more than doubled when compared to multiparous women, but reached statistical levels only for uncertified midwives (Certified midwives: 17.6/10,000 for nulliparous versus 7.3/10,000 for multiparous; RR 2.42, 95%CI: 0.92–6.37. Uncertified midwives: 23.7/10,000 for nulliparous versus 10.6/10,000 for multiparous; RR 2.22, 95% CI: 1.31–3.75). Neonatal mortality rates at home births for women ≥41 weeks versus those <41 weeks were significantly doubled for uncertified midwives (21.6 versus 10.4/10,000; RR 2.06; 95%CI: 1.23–3.44) but not for certified midwives (10.3 versus 9.8/10,000; RR 1.06, 95%CI 0.38–2.98). Neonatal mortality rates for women ≥41 weeks were doubled for uncertified midwives when compared to certified midwives but did not reach significant levels (21.6 vs 10.6/10,000; RR 2.08, 95%CI: 0.8–5.4).

## Comments

This study confirms that when compared to midwife-attended hospital births, neonatal mortality rates at home births are significantly increased. While NNM was increased in planned homebirths attended by uncertified midwives when compared to certified midwives, this difference was not statistically significant. Neonatal mortality rates at home births were not significantly different in relationship to professional certification status of the birth attendant, whether the delivery was by a certified or an uncertified birth attendant.

## Clinical Implications

There is a clear pattern in our study: Protection against the significantly increased risk of neonatal mortality of home birth generally, for all women, for nulliparous and multiparous women, and for pregnancies <41 and ≥41 weeks gestation is not provided by certification by the American Board of Midwives. The increased neonatal mortality rates at home births are more strongly associated with the location of the birth at home rather than the birth attendant’s professional certification. Considering the location of the births at home with the unpredictability of adverse events and inability to perform expeditious interventions such as cesarean deliveries, it’s speculative whether attendance by certified physicians provides sufficient protection.

## Strength and Weakness

The strength of our study is that we used the linked birth/infant death data set (period linked file) which is generally the preferred source for infant and neonatal mortality in the United States.[12] There are some limitations in our study. As in our previous study,^7^ our results likely underestimate the actual neonatal mortality rates in home births, because the neonatal outcomes for patients transferred from home to the hospital are counted in the CDC linked data as hospital and not home birth neonatal outcomes. On the 2003 revised US birth certificate, information on planned and unplanned home birth is collected inconsistently, but information on whether a birth in the hospital is the result of a transferred home delivery is not collected. Further data may be helpful to reevaluate these findings for more recent years.

## Conclusions and Implications

Patients considering a home birth should understand that choosing a home birth attendant by professional certification status, whether it’s a certified or uncertified midwife, does not significantly improve neonatal mortality when compared to planned hospital births which has significantly lower neonatal mortality rates when compared to planned home births. Studies from Europe have shown that out-of-hospital birth can be a safe option in low-risk women. [14,15] whereas hospital births in the US by either midwives or physicians are safer than planned home births by certified or uncertified midwives. In response to expressions of interest in planned home birth by pregnant women, obstetric providers should explain the clinical reality of increased neonatal mortality at home births and recommend strongly against planned home births and strongly recommend for planned hospital births. They should explain that these recommendations are based on the documented increased risk of neonatal mortality in home births that is not altered by the professional certification status of the home birth attendant, and is unlikely to improve even with attendance by certified physicians because of the unavailability of resources and delay in interventions at home. Providing evidence-based recommendations is essential for obstetric providers to fulfill their professional responsibility and to empower the autonomy of pregnant women in the informed consent process by providing clinically important, evidence-based information. [16,17,18]

Obstetricians and other concerned professionals should understand, identify, and address the root causes of planned home births. They should respond to expressions of interest and motivations for planned home birth through the informed consent process with evidence-based recommendations against it, improve hospital settings and consider in-hospital birthing centers, address obstetric interventions, and provide excellent and compassionate hospital emergency obstetric care to women transported from planned home birth.[16,17,18]
